# Enterococcus lactis is ecologically and genetically distinct from the major opportunistic pathogen Enterococcus faecium

**DOI:** 10.1099/mgen.0.001420

**Published:** 2025-06-06

**Authors:** Theodor A. Ross, Jessin Janice, Sergio Arredondo-Alonso, Iren H. Löhr, Einar Holsbø, Jukka Corander, Anna K. Pöntinen, Michael Kampffmeyer, Kristin Hegstad

**Affiliations:** 1 Department of Physics and Technology, UiT The Arctic University of Norway, Tromsø, Norway; 2 Centre for New Antibacterial Strategies, UiT The Arctic University of Norway, Tromsø, Norway; 3 Norwegian Centre for Detection of Antimicrobial Resistance, Department of Microbiology and Infection Control, University Hospital of North Norway, Tromsø, Norway; 4 Research Group for Host-Microbe Interactions, UiT The Arctic University of Norway, Tromsø, Norway; 5 Section for Development, Department of Microbiology, Oslo University Hospital, Oslo, Norway; 6 Department of Biostatistics, Faculty of Medicine, University of Oslo, Oslo, Norway; 7 Parasites and Microbes, Wellcome Sanger Institute, Hinxton, Cambridgeshire, UK; 8 Department of Medical Microbiology, Stavanger University Hospital, Stavanger, Norway; 9 Department of Clinical Science, Faculty of Medicine, University of Bergen, Bergen, Norway; 10 Department of Computer Science, UiT The Arctic University of Norway, Tromsø, Norway; 11 Helsinki Institute of Information Technology, Department of Mathematics and Statistics, University of Helsinki, Helsinki, Finland; 12 Department of Genetics, University of Cambridge, Cambridge, UK

**Keywords:** *Enterococcus lactis*, *Enterococcus faecium*, evolution, genomics

## Abstract

*Enterococcus faecium* is a major human opportunistic bacterial pathogen and a close relative of the recently established species *Enterococcus lactis*. As a species, commensal *E. lactis* remains relatively understudied, and its genetic connectivity with *E. faecium* is not thoroughly understood. Here, we introduce a large collection of whole-genome sequenced isolates comprising 894 *E. faecium* and 392 *E. lactis* genomes. Using these genomes to complement publicly available data, we studied the genome content and the evolutionary relationship between these species. A wider range of host species was observed in *E. faecium*; in particular, there is a radiation of *E. faecium* clades specialized to domesticated and pet animals among which *E. lactis* is uncommon. Of note, pangenome analyses reveal that *E. lactis* has significantly more allelic variation and lower recombination rates in core genes compared with *E. faecium*. These observations suggest that *E. lactis* represents a population that has occupied its ecological niche longer than *E. faecium* has. This study enhances understanding of the evolutionary histories of these species and highlights the importance of sampling and studying closely related commensal bacteria in addition to clinically relevant opportunistic pathogens.

**Impact Statement**

To our knowledge, this work is the first to perform a broad-scale population analysis with several hundred *Enterococcus lactis* whole-genome sequences. We analyse this study population and compare it to a globally distributed *Enterococcus faecium* population. In doing so, we highlight how these populations differ in virulence, antimicrobial resistance, average nt identity, plasmid content and horizontal gene transfer dynamics as well as reinforce the assertion that they belong to different species designations. Through these comparisons, we find potential marker genes that could be used in differentiating *E. lactis* from *E. faecium* as well as evidence that *E. lactis* has occupied its niche longer than *E. faecium* and the *repUS15* (repA_N) megaplasmid can transmit genetic material between these species.

## Data Summary

Detailed description of the genome collection and relevant accession numbers are available in Dataset S1 (available in the online Supplementary Material).

## Introduction

*Enterococcus faecium* is a Gram-positive gut commensal and major opportunistic pathogen in many mammals, including humans [1,2]. The clinical relevance of *E. faecium* has increased in the last decades and is a result of its ability to cause infections as well as its antimicrobial resistance (AMR) profiles [3]. In addition to innate resistance to several antimicrobial compounds, *E. faecium* can acquire new resistance genes through horizontal gene transfer (HGT) [4,6]. This, coupled with the ability to survive in hostile conditions encountered in healthcare settings, makes *E. faecium* a common cause of nosocomial infections [7].

Previous population studies describe *E. faecium* as being composed of two major subpopulations named clades A and B [4,8,11]. These studies divide clade A into healthcare-associated clade A1 and healthy human and animal-associated non-A1 lineages. Clade B is described as community associated [4,8,12]. Consequently, most genomic and epidemiological studies have focused primarily on the clinically relevant clade A1 population of *E. faecium*.

It was recently established that the population previously called clade B *E. faecium* belongs to the species designated *Enterococcus lactis* [13]. The taxonomic separation of these species was later further supported using average nt identity (ANI) values between 128 *E. faecium* and 64 *E. lactis* genomes [14]. *E. lactis* was originally isolated from milk and was noted as being closely related to *E. faecium* [1]. Historically associated with food products and rarely related to infection, *E. lactis* is comparatively undersampled in studies relative to *E. faecium*. Pangenome analysis has shown significant differences between these two species in gene content, including gene clusters involving metabolic pathways for specific carbon compounds [14] as well as sufficient allelic differentiation of *gluP* between the species to be used as a genomic marker to differentiate them [15].

The evolutionary relationship between these two species remains relatively poorly characterized. Previous studies estimate the divergence of *E. faecium* and *E. lactis* to have occurred ~2,800 years ago [8,9]. These values were based on study populations which included at most 37 *E. lactis* isolates and do not explain potential driving factors or ancestral relations.

A thorough genomic comparison between these two closely related species requires a more balanced sampling of the two populations than has been achieved in previous studies. Many of them focus primarily on clinically relevant subpopulations, resulting in a gap of knowledge when it comes to *E. lactis*. Our novel dataset of 1,286 whole-genome sequences from Norwegian samples, including clinical (*n*=577), commensal (*n*=561) and marine (*n*=148) isolates, together with publicly available whole-genome sequences (*n*=1,706), allows us to conduct the first in-depth population-wide analysis of the relationship between *E. faecium* and *E. lactis*.

## Methods

### Data collection

We utilized a collection of 3,007 whole-genome sequences of *E. faecium* and *E. lactis*. After filtering the population to remove sequences with large mean Levenshtein distances between core gene sequences, 2,992 genomes were kept for analysis. These included a novel collection of 1,286 Norwegian isolates, 1,117 isolates from Arredondo-Alonso *et al*. (*n*=1,060 short read, *n*=62 hybrid assemblies) [4], 215 from Rios *et al*. [9], 73 from Lebreton *et al*. [8] and 301 circularized sequences from the National Center for Biotechnology Information (NCBI) (*n*=294 *E*. *faecium*, *n*=7 *E*. *lactis*) (downloaded on 17 February 2023). The Norwegian strains were primarily sequenced using Illumina short-read sequencing technology. The collection was subsequently refined by resequencing 126 isolates with a long-read Pacific Biosciences (PacBio) NGS platform. Isolates were selected for long-read sequencing to evenly represent all groups within the population’s phylogeny. All overlapping sequences between different groups were removed, retaining any circularized genomes. The combined study population contained a total of 492 genomes that were fully circularized or sequenced with long-read sequencing technology. Detailed description of the genome collection and relevant accession numbers are available in Dataset S1.

The Norwegian isolates came from different collections: (1) commensal isolates (*n*=500) (this study) from a representative adult population in Norway [16], (2) marine isolates (*n*=148) from bivalve batch samples collected along the Norwegian coast [17], (3) a random subset of vancomycin-resistant isolates (*n*=224) from the Norwegian surveillance system for communicable diseases and vancomycin-susceptible invasive isolates (*n*=266) from the national surveillance system for AMR in microbes (the Norwegian VRE study) [18] and (4) vancomycin- and/or linezolid-resistant *E. faecium* (*n*=148) collected at the Norwegian Centre for Detection of Antimicrobial Resistance (K-res).

Commensal enterococci from the Tromsø7 collection were collected by screening faecal samples from 2,962 participants in the Tromsø7 survey [16] for the presence of *E. faecium/E. lactis* and using agar based on *E. faecium* HiCrome™ Agar Base and *E. faecium* selective supplement (Sigma-Aldrich, Saint-Louis, MO, USA). *E. faecium/E. lactis* were isolated from 500 different participants in this representative adult (40–85 years) population from Norway.

### Species identification and antimicrobial susceptibility testing

The Norwegian isolates were identified by MALDI-TOF MS according to the manufacturer’s protocol (Bruker Daltonics, Bremen, Germany). The applied Bruker library [MALDI Biotyper® Compass Explorer (v2020)] was unable to distinguish *E. faecium* from *E. lactis*.

In addition, the Norwegian isolates were tested for phenotypic resistance to ampicillin, vancomycin, gentamicin and linezolid using the Sensititre™ EUENCF panel (Thermo Fischer Scientific, Waltham, MA, USA) or for the Tromsø7 and Norwegian VRE study collections as described in Al Rubaye *et al*. [18]. The antimicrobial susceptibility test (AST) data were interpreted according to the European Committee on AST (EUCAST) clinical breakpoints [19,19].

### DNA isolation and whole-genome sequencing

Initially, all the 1,286 Norwegian samples within this study were subjected to short-read sequencing. DNeasy Blood and tissue kit (Qiagen, Hilden, Germany) or the MagNA Pure 96 DNA and Viral NA Small Volume Kit was used to extract genomic DNA and Qubit fluorometer (Invitrogen) to quantify the concentration of total genomic DNA. Most of the samples were sequenced at the Genomic Support Center Tromsø using Illumina NextSeq 550 paired-end platform as described previously [20], while the Tromsø7 collection samples were sequenced on a MiSeq platform using 2×300 bp read length.

A subset of 126 isolates was subsequently long-read sequenced as reference genomes and was selected to cover the genetic variation within the population-wide phylogeny. The EZ1 Advanced XL (Qiagen) automated system and subsequent purification using AMPure XP beads (Beckman Coulter, Brea, CA, USA) were used to extract DNA for long-read sequencing. The genomic DNA quality and concentration were assessed with gel electrophoresis, Nanodrop and Qubit fluorometer. Long-read sequencing was performed on Pacific Biosciences Sequel II at the Norwegian Sequencing Centre (University of Oslo) as described in Al Rubaye *et al*. [18]. The resulting PacBio read length ranged from 10 to 20 kb.

### Genomic assembly

For Illumina sequenced samples, SPAdes v3.12.0 [21] was used for genome assembly. A cut-off maximum of 400 contigs and minimum of 40× genome coverage was used to consider the assemblies as eligible to be included in the analyses. Moreover, the genome size should not show more than ±10% fluctuation compared with the smallest and biggest complete *E. faecium* or *Enterococcus faecalis* genome assemblies in the NCBI’s RefSeq database. For PacBio-sequenced samples, Unicycler v0.4.9 [22] was used for assembly. The assemblies that Unicycler was unable to circularize were reassembled using Canu v1.4 [23].

Short reads collected from the Arredondo-Alonso study population were assembled using Shovill v1.1.0 based on SPAdes v3.15.5 [24,25]. This collection of assemblies was then filtered to remove those with more than 600 contigs and a median coverage below 20×. The quality control thresholds for this collection were intentionally more lenient to retain as many samples as possible.

### Average nt identity

The ANI values were computed between all pairwise combinations of sequences using fastANI (v1.33) with default parameters to compare between *E. faecium* and *E. lactis* to a 95% speciation threshold [26].

### Phylogeny and population clustering

Multiple sequence alignments were computed using Snippy v4.6.0 using default parameters [27]. The *E. faecium* population was aligned to the NCBI reference sequence Aus0004. At the time of analysis, the *E. lactis* reference sequences available on the NCBI were agricultural or dairy isolates. To better represent *E. lactis* as it is present in human gut microbiota, one of the PacBio sequenced Norwegian isolates was chosen as an *E. lactis* reference: sequence 50993357.

The multiple sequence alignments produced with Snippy were used to estimate the maximum likelihood phylogenies of each species. This was executed using RAxML-NG v4.6.0 with default parameters [28]. A general time-reversible phylogeny for the combined population was estimated using FastTree v2.1.11 from a combined nt alignment [29]. These phylogenies were not computed by masking regions of recombination. Using Gubbins [30] to detect recombination in these populations masked over 70% of each genome as recombination regions. Reconstructing maximum likelihood phylogenies with such large gaps in the alignments failed. The combined population phylogeny and ribosomal multilocus sequence typing (rMLST) predictions were used to determine the species designation of each isolate [31,32].

The multiple sequence alignments generated with Snippy were used for clustering the combined population, as well as the species-specific populations, and were clustered using Bayesian population clustering implemented in fastbaps v1.0.8 [33]. After dividing the study population into subpopulations, the Shannon diversity index (DI) was used to compute the relative diversity of isolation sources for each population [34].

### Pangenome estimation

The genome assemblies were annotated using Prokka v1.14.6 [35]. The pangenomes of the combined study population, as well as individually for *E. faecium* and *E. lactis* subpopulations, were subsequently estimated using Panaroo v1.3.4 [36] in moderate mode with merging paralogues and removing invalid genes. Panaroo was also used to produce alignments for each cluster of orthologous gene (COG) detected within the study population. Thresholds for core, soft core, shell and cloud genes used in this study are the same as those used by the authors of Panaroo. Core genes were considered intrinsic to their respective populations.

### Population filtering using core gene alleles

After the pangenome of the combined population was estimated, the core genes in each isolate were further analysed. For each core gene, the Levenshtein distance was computed between the alleles found in all unique pairs of genomes [37]. A running average was used to compute the mean core gene distance for each pair of sequences across all core genes. The resulting distance matrix was analysed for columns that represented outliers from the rest of the population.

Using this method, 15 sequences were removed from the population, 3 were circularized *E. faecium* assemblies downloaded from the NCBI, 5 were short-read assemblies collected from Arredondo-Alonso *et al*. and 7 were from data shared by Rios *et al*.

### Core gene allelic diversity analysis

A relative allelic DI was computed for each gene independently following a procedure similar to what is used by White *et al*. [38]. This study divides the number of unique alleles by the total number of times a gene is found within a respective population as shown in equation 1. This allows us to account for genes that appear multiple times in one sequence more robustly.


(1) $$DI=\frac{\#\;distinct\;alleles}{\#\;gene\;instances}$$

These DI values were computed independently for each core gene as they appeared within each species and clade subpopulation. For this purpose, core genes were defined as being present in over 99% of isolates in a given population.

### Inference of HGT using consistency indices

Consistency indices (CIs) offer a way to approximate homoplasy by comparing genetic alignment information to a phylogenetic tree. CI values closer to zero indicate more homoplasy in the query alignment. Homoplasy is often assumed to be caused by HGT, meaning CI values can be used as an estimate of relative rates of HGT rates.

The alignments produced by Panaroo and the maximum likelihood phylogenies produced with RAxML-NG were used to estimate the CIs. The R package Phangorn v2.11.1 was used to compute the CI values [39]. For each subpopulation analysed, the population-specific core genome and phylogenies were used for the approximation.

### Estimation of gene gain and loss events in accessory genes

The estimated pangenomes and approximate maximum likelihood phylogenies were used to approximate gene gain and loss events using Panstripe v0.3.1 [40]. The model was implemented assuming a Gaussian distribution while including an intercept term. The combined phylogeny computed with FastTree was partitioned into relevant subpopulations for this analysis. This tree was used following suggestions from Panstripe documentation to guarantee a consistent scale for the trees used to fit each model.

### Plasmidome analysis

Mlplasmids v2.1.0 was used to predict whether contigs are chromosome or plasmid derived [41]. The mlplasmids toolbox includes a model pre-trained on *E. faecium* which was applied to the *E. faecium* sequences used in this study. Since there was no model for *E. lactis*, we trained a new model using the same methodology described in the mlplasmids publication [41]. The mlplasmids models were not used to predict associations for contigs shorter than 1,000 bp, as recommended by the package authors.

A total of 67 *E. lactis* isolates sequenced with PacBio technology and assembled using Unicycler v0.5.0 [22] were used to train our new *E. lactis* mlplasmids model. Circular Unicycler contigs were labelled as plasmids if they were shorter than 350 kb in length; otherwise, they were labelled as chromosomes. SPAdes contigs for the corresponding sequences were labelled as plasmid or chromosome associated by mapping them to labelled Unicycler contigs with bwa-mem v2.2.1 and samtools v1.17 [42,43]. After this process, the 67 Unicycler assemblies contained 176 labelled, circular contigs. These corresponded to 8,007 mapped and labelled SPAdes contigs. Eight *E. lactis* strains were excluded from training and reserved for model evaluation. This resulted in 7,313 labelled SPAdes contigs for model training and 694 to be used for validation. The eight *E. lactis* strains set aside were chosen to include five randomly chosen samples containing at least one plasmid and three randomly chosen which contain zero plasmids. These were chosen to provide both positive and negative controls while also matching the plasmid frequency in the training dataset.

The sequences designated for training were again split into a training set, containing 5,850 contigs, and a test set, containing 1,463 contigs. Pentamer frequencies were extracted from each contig to be used as feature vectors. Each of these pentamer frequency vectors was used to train and test five types of binary classification models: logistic regression, naïve Bayes, decision tree, random forest (RF) and kernel support vector machine (SVM) classifiers all implemented with the mlr v2.19.1 package in R [44]. All models were trained using 10-fold cross-validation randomly selected across the 5,850 training samples. This procedure was used to perform a grid search over the hyperparameters of the decision tree, RF and SVM models. The grid search was performed to select the hyperparameters that optimize the mean misclassification error. The trained models were then evaluated using the 694 withheld validation contigs. They were also tested against the withheld contigs that were longer than 1,000 bp (*n*=416 contigs). Like the original mlplasmids models, the SVM was identified as the most accurate model for prediction with *E. lactis* contigs.

Specific plasmid replicons were also screened for using PlasmidFinder v2.1.6 [45]. The screened replicons were filtered to have a minimum coverage and identity thresholds of 90%.

### Resistance and virulence factor screening

The resistome and virulome of the study population were studied by screening the assembled genomes for known resistance and virulence factors. AMRFinderPlus (v3.11.4) was used for detecting AMR factors [46]. A collection of experimentally verified virulence factors in *E. faecium* was used to define a custom dataset for screening using ABRicate (v1.0.1) using minimum coverage and identity thresholds of 90% [47]. This curated list is the same as the one used by Al Rubaye *et al*. and contains sequence information of 30 unique virulence factors [18].

### Recombination analysis

A sub-selection of the study population was chosen to try to detect recombination events across the species boundary. For this, we isolated the entire *E. lactis* population and the *E. faecium* genomes with the high ANI when compared with *E. lactis*. A multiple sequence alignment was generated for this selection using Snippy v4.6.0 and analysed for recombination using Gubbins v3.3.5 [27,30]. The ‘filter percentage’ parameter in Gubbins was raised to 40 to ensure all of the desired isolates were retained in the analysis. The predicted recombination events were then screened for any that crossed the species boundary.

### Statistical analyses

Hypothesis tests comparing the diversity and CI value distributions of various subpopulations were computed using the Mann–Whitney U test implemented in scipy v1.12.0 [48].

*P*-values reported for gene exchange rates in the accessory genome and for comparing virulence and AMR factors were computed with permutation tests using 1,000 bootstrap replicates of the pangenome model parameters.

### Data availability

Sequence data generated within this study have been deposited at the ENA with study accession numbers PRJEB71064 and PRJEB71065 (Tromsø7 collection) and PRJEB64173 (K-res collection). Interactive view and descriptive data on the 2,992 *E. faecium* and *E. lactis* isolates are publicly available within the Microreact project https://microreact.org/project/lactis-faecium-population-study. The *E. lactis* mlplasmids model has been integrated in the package https://gitlab.com/sirarredondo/mlplasmids. Available metadata for the isolates used in this study as well as the download information for publicly available sequences collected are available in Dataset S1.

## Results

### Population structure and ANI describe a deep phylogenetic split in *E. lactis*

The combined midpoint-rooted maximum likelihood phylogeny of 2,992 genomes displays a clear separation of 2 groups corresponding to *E. lactis* and *E. faecium* (Fig. S1). The division of these two species is also supported by ANI analyses. The mean inter-species ANI of 94.59% is significantly below the 95% speciation threshold proposed by Jain *et al*. (*P*<10^−15^, *T*=−2,630.82, Degrees of Freedom (DoF)=2,304,503), agreeing with previous studies [26]. The mean intra-species ANI values of 99.03% in *E. faecium* and 98.44% in *E. lactis* were both significantly greater than 95% (*E. faecium*: *P*<10^−15^, *T*=28,592.80, DoF=6,441,443) (*E. lactis: P*<10^−15^, *T*=2,350.87, DoF=206,115) (Fig. S2). These ANI values derived from hundreds of *E. lactis* and thousands of *E. faecium* genomes reinforce the separation of these two closely related species.

**Fig. 1.**
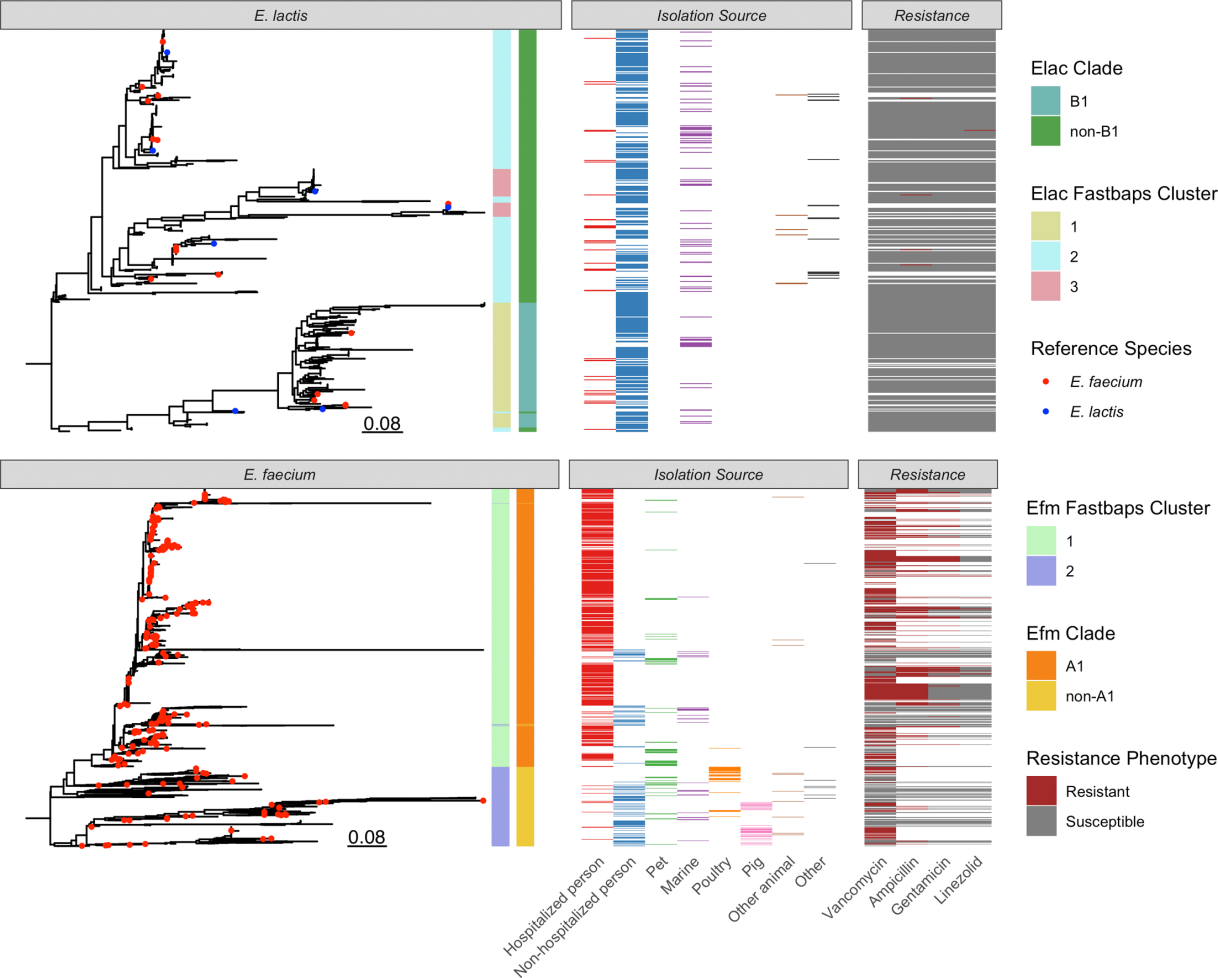
Midpoint-rooted maximum likelihood phylogenies of the *E. lactis* (*n*=454) (top) and *E. faecium* (*n*=2,538) (bottom) populations. Population clusters as determined with fastbaps were computed independently for each species and are displayed in the tree panels. The coloured leaf tips correspond to circularized assemblies collected from the NCBI. The isolation source and phenotypic AMRs are displayed for all isolates that had such data available. Elac = *E. lactis*; Efm = *E. faecium*.

Midpoint-rooted maximum likelihood phylogenies of 2,538 *E. faecium* and 454 *E. lactis* isolates both display splits between major groups in their respective populations (Fig. 1), whereas the combined phylogeny does not depict the separation of these intra-species clades as clearly (Fig. S1). As shown by the coloured leaf tips in the *E. lactis* phylogeny, all of the *E. lactis* reference sequences collected from the NCBI fell within the *E. lactis* population in this analysis. In contrast, 19 of the NCBI reference sequences were incorrectly labelled as *E. faecium* and actually belong to *E. lactis*.

The population clusters computed with hierarchical Bayesian analysis (fastbaps) closely reflect the large divisions in the phylogenetic structure of both *E. faecium* and *E. lactis* (Fig. 1). Each species shows at least two major fastbaps clusters, which were used to distinguish the major clades in *E. faecium* and the *E. lactis* clades described within this study. *E. faecium* fastbaps cluster 1 closely aligns with clinically associated clade A1. As a result, we use this cluster label to define clade A1 for further analyses, while the remaining isolates are termed non-A1.

The *E. lactis* population contains a sub-group that consists of tightly grouped isolates with similar branch lengths corresponding to *E. lactis* fastbaps cluster 1. This group appears to share a relatively recent ancestor node in the *E. lactis* phylogeny and define a clearly visible clade (Fig. 1 top). For the purposes of this study, this will be designated as clade B1, with the rest of the *E. lactis* population consisting of non-B1 isolates.

The species designations were also predicted for each isolate using rMLST. Five of the rMLST predictions did not match the phylogeny-based species labels. Two were considered non-B1 *E. lactis* by the phylogeny, but rMLST found equal support for these genomes to be classified as *E. lactis* or *E. faecium*. Two A1 *E. faecium* isolates, according to the phylogeny, were labelled as *E. faecalis* and *Enterococcus avium* by rMLST. For both of these examples, the second most supported species was *E. faecium*. The final disagreement is a non-B1 *E. lactis* isolate, which rMLST determined is *E. faecium*. With only five of these conflicting species designations, the alignment between rMLST and the core genome phylogeny-based labels is very high. The disagreements are likely due to the low number of *E. lactis* isolates in the rMLST database at only 250 at the time of writing, which is far fewer than the 19,706 *E. faecium* isolates.

The ANI distributions show several peaks within the *E. lactis* population, in contrast to the almost uni-modal *E. faecium* intra-species ANI distribution. The ANI distribution between B1 and non-B1 *E. lactis* has the lowest mode of the intra-species distributions (Fig. S3), indicating greater genomic distance between the two subpopulations when compared with A1 and non-A1 *E. faecium*.

### Isolation source diversity correlates with phylogenetic diversity

**Fig. 2.**
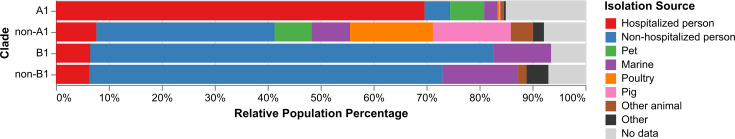
Relative distributions of isolation sources that make up each population group in the combined study population.

*E. lactis*, while being sourced primarily from non-hospitalized humans, also contains marine, human clinical and animal samples. Of the 1,439 hospital-associated samples, 29 were *E. lactis* – 9 clade B1 and 20 non-B1 isolates (Fig. 2).

Non-A1 *E. faecium* displays the most diverse set of isolation sources (Shannon DI of 1.924 natural digits) being roughly twice as diverse as A1 (1.037) and non-B1 *E. lactis* (1.107). Clade B1 is the least diverse (0.801). The high level of isolation source specificity in *E. faecium* clade A1 is reflected in its phylogenetic structure as a clearly separated subpopulation with relatively short branches. Conversely, non-A1 *E. faecium* has a wider diversity of isolation sources and shows more intra-clade phylogenetic divergence (Fig. 1). This relationship is mirrored when comparing B1 and non-B1 *E. lactis*, as non-B1 *E. lactis* has both higher source and phylogenetic diversity (Fig. 1). Consequently, we see a positive relationship between genomic diversity observable in the phylogeny and the isolation source diversity of each subpopulation.

### Core genome divergence highlights differences in metabolic pathways between *E. faecium* and *E. lactis*

Comparing pangenomes reveals that each species contains its own set of species-specific core COGs relative to each other (Table 1). The *E. lactis* population has fewer total unique COGs, but a higher ratio of core-to-mean COGs than *E. faecium*.

**Table 1.** Estimated pangenome sizes of the combined and species-specific populations measured in clusters of genes (COGs)

Population	Total	Core	Mean	Core: mean	Unique	Unique core
Combined	16,397	1,625	2,661.16	0.6106	–	–
*E. faecium*	14,983	1,699	2,678.90	0.6342	7,719	18
*E. lactis*	8,620	1,764	2,561.95	0.6885	1,356	8

Core COGs are determined to be those present in over 99% of the respective population.

The unique *E. lactis* core genes include *coiA* (competence protein), *xly* (xanthan lyase) and six genes annotated as hypothetical proteins. The 18 unique core COGs in *E. faecium* include 9 genes labelled as hypothetical proteins and 9 annotated COGs involved in metabolism, transport and regulation (Section S2, Dataset S2).

**Fig. 3.**
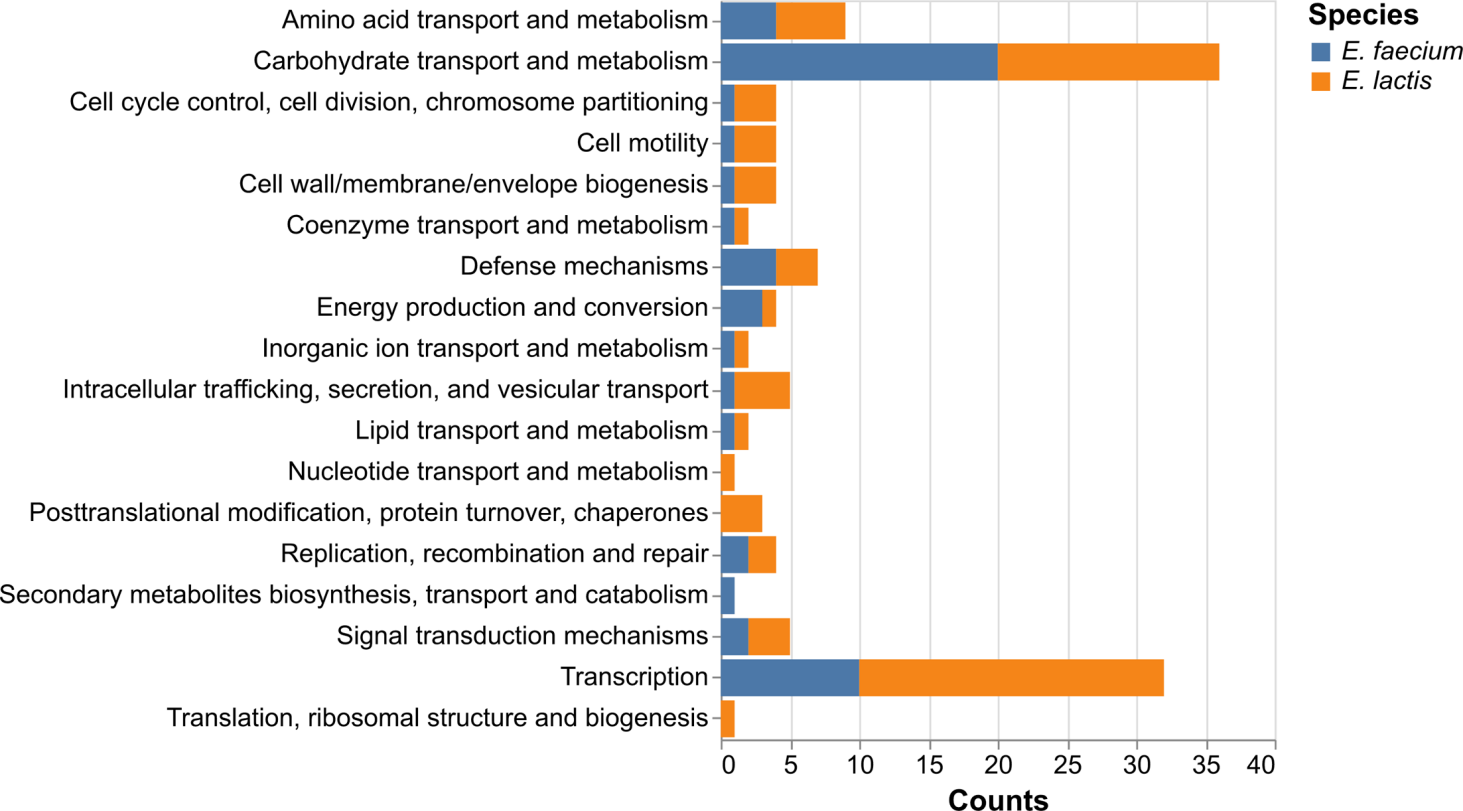
Functional categories of COGs that are in the soft core (>95%) in one species and cloud (<15%) in the other. The colours correspond to the species in which the genes are commonly found. Hypothetical proteins of unknown function were not included in this chart.

Using the pangenome matrix, we isolated the COGs that were highly present in one species (soft core genes) while being rare in the other (cloud genes). These thresholds flagged 77 COGs predominantly found in *E. faecium* and 100 COGs found in *E. lactis*, respectively. A large number of these COGs were associated with carbohydrate and aa metabolism for both *E. lactis* and *E. faecium*, indicating divergent metabolic pathways for the two species (Fig. 3). The second most frequently flagged categories of genes are transcription regulation genes, which likely correspond to the regulation of the non-overlapping core genes. These results align with previously reported observations that carbohydrate metabolism-associated COGs vary between *Enterococcus* species more than other observed functional categories [49].

### *E. lactis* core genome displays more allelic diversity and less homologous recombination than *E. faecium*

**Fig. 4.**
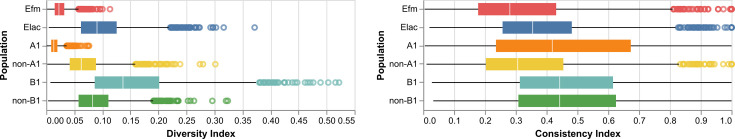
Tukey box plots displaying the diversity (left) and consistency (right) indices of the core COGs in each population group. Efm, *E. faecium*; Elac, *E. lactis*.

Allelic DIs were computed for all core genes present in each species and clade to represent the relative allelic variation. This analysis of core genes in each species shows that *E. lactis* has a significantly more diverse core genome than *E. faecium*, in terms of allelic diversity (*P*<10^−15^, *U*=2.806×10^6^) (Fig. 4 left), implying a less clonal population structure in *E. lactis*.

Clade A1 had the lowest core genome allelic diversity, significantly lower than the non-A1 (*P*<10^−15^, *U*=1.673×10^5^), suggesting that clade A1 has the most clonally evolving core genome across the considered groups, which aligns with the observations of successful hospital clones [50]. B1 *E. lactis* displays the most diverse core genome with a mean DI value of 0.1522 (sd=0.0877).

CIs were also computed for all core genes in each species and clade subpopulation. The CI measures how closely the allelic variation of a gene corresponds to the phylogeny in order to identify homoplasies. In the context of core genes, these homoplasies are assumed to be caused by homologous recombination events.

*E. lactis* displays significantly higher CI values than *E. faecium* (*P*=1.424×10^−31^, *U*=2.550×10^6^) (Fig. 4 right). The higher CI values in *E. lactis* indicates lower rates of recombination in the core genes, despite the higher allelic diversity observed in the same set of core genes.

Comparing CI distributions from individual clades shows that the core genome of A1 *E. faecium* has significantly higher CI values than non-A1 *E. faecium* (*P*=3.767×10^−39^, *U*=2.523×10^6^). This indicates that the core genome of A1 *E. faecium* is significantly less influenced by homologous recombination, relative to non-A1 *E. faecium*. This aligns with previously reported evidence that A1 *E. faecium* shows scarce recombination between different clonal strains [51]. Non-B1 *E. lactis* and clade B1 isolates did not show significantly different CI distributions (*P*=0.272, *U*=2.041×10^6^), indicating similar levels of recombination in their core genomes.

### Accessory gene-associated HGT rates are higher in *E. faecium* than in *E. lactis*

**Fig. 5.**
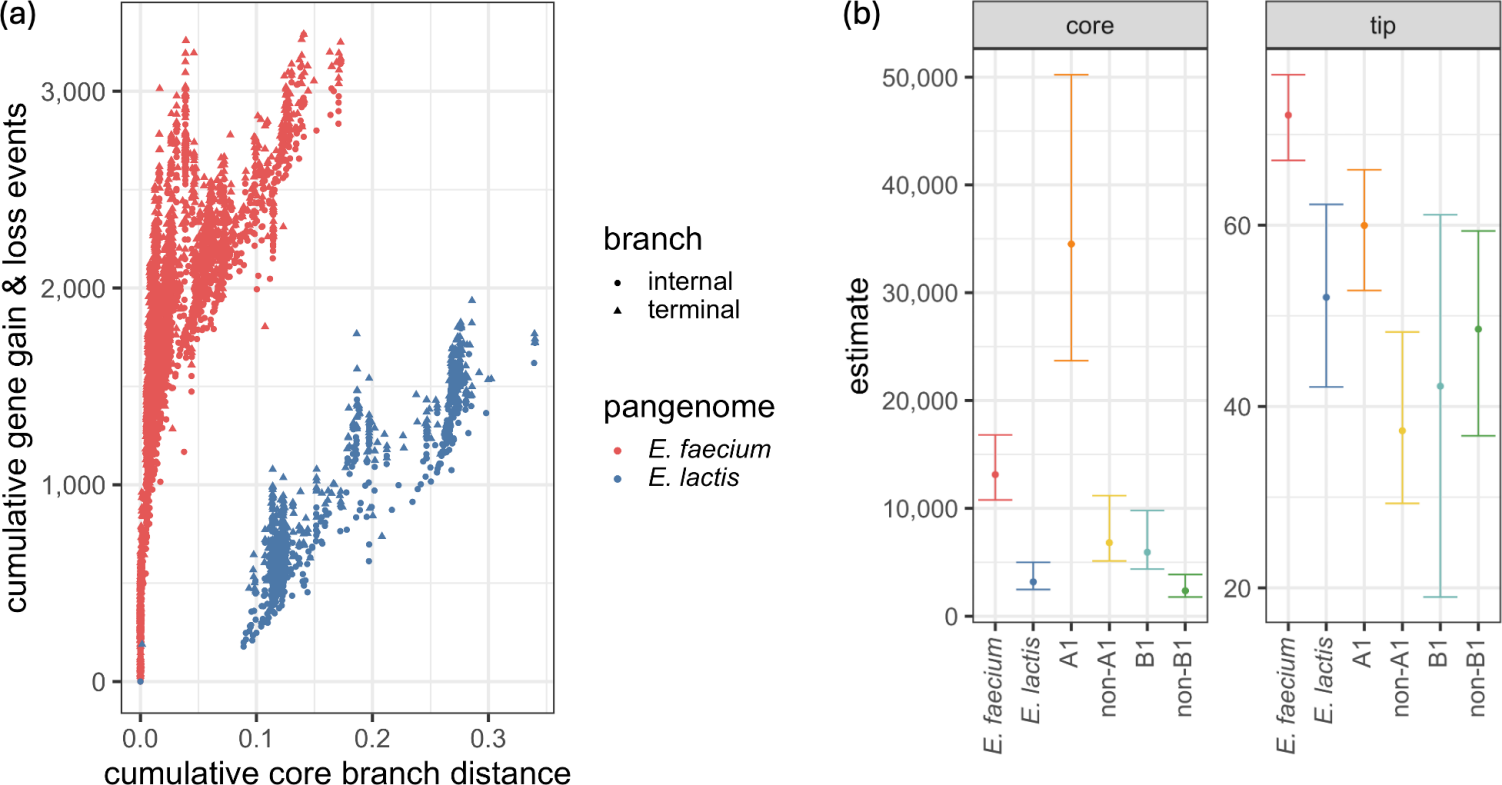
Summary of models produced with Panstripe using the estimated pangenome and a combined population phylogeny computed with FastTree. (**a**) Estimated gene gain and loss events computed by Panstripe. (**b**) Parameters of the gene exchange models fitted to each clade. The ‘core’ parameter describes the association between gene gain and loss events with core phylogeny branch length. The ‘tip’ parameter describes gene exchange events that occur only at the tips of the phylogenies.

Gene exchange rates estimated with Panstripe are shown in Fig. 5. Each clade was analysed independently to compare the behaviour of different subpopulations. The *E. faecium* and *E. lactis* populations were observed to have significantly different rates of gene exchange across the accessory genome (*P*=0.001) with *E. faecium* having larger HGT rates (Fig. 5b). This difference is clearly visible in the cumulative total gene gain and loss events as well (Fig. 5a). The estimated population models further indicate that the gene exchange rates of clade A1 are significantly different from the rates of non-A1 (*P*=0.001), with clade A1 showing the highest rate of accessory gene-associated HGT of all clades. Clade B1 and non-B1 *E. lactis* also showed significantly different rates of gene exchange (*P*=0.001). Additionally, non-A1 *E. faecium* was observed to have statistically different gene exchange rates from the *E. lactis* population (*P*=0.005).

The estimated population models also approximate gene presence and absence rates observable only at the tips of the phylogeny (Fig. 5b). Significant differences in this parameter indicate differences in short-lived, highly mobile genetic elements (MGEs) in the respective populations. The rates of these short-lived MGEs in the *E. faecium* and *E. lactis* phylogenies were significantly higher in *E. faecium* (*P*=0.001). Clade A1 was observed to have higher MGE presence rates than non-A1 (*P*=0.001). Clades B1 and non-B1 did not show significantly different rates of MGE presence (*P*=0.588). Comparing non-A1 *E. faecium* to the entire *E. lactis* population did not show a significant difference in MGE presence rates (*P*=0.056).

### *E. lactis* contains one more intrinsic resistance factor than *E. faecium* but fewer acquired resistance and virulence factors

Genome screening for AMR genes using AMRFinderPlus confirms that *E. faecium* tends to contain significantly more resistance factors than *E. lactis* (*P*=0.0001). This result primarily reflects the high prevalence of acquired resistance factors in *E. faecium*, particularly in clade A1, which aligns with previous studies [8,10]. Despite this, *E. lactis* contains one more intrinsic (present in >99% of isolates) resistance factor than *E. faecium* does. Both species contain *copB*, *msr(C)* and *aac(6′)-I* as chromosomally associated intrinsic resistance factors, but *E. lactis* also contains the pleuromutilin resistance conferring *eatA* (T450I) mutation as a core resistance factor (Fig. S4).

Screening for virulence factors was done via blast search using a custom database of confirmed virulence factors in enterococci [18] (Fig. S5). On average, *E. faecium* contains more of these known virulence factors than *E. lactis* (*P*=0.0001). Comparing *E. lactis* subpopulations shows that clade B1 contains more virulence factors than non-B1 isolates do (*P*=0.0019). *E. faecium* and *E. lactis* shared several intrinsic virulence factors: *ccpA*, *fnm* and *lysM4*. Additional factors *bepA* and *lysM2* were intrinsic in *E. faecium*, whereas the *gls20*, *gls33* and *glsB1* genes were intrinsic in *E. lactis*. Both species contained several more virulence factors that are considered soft core (Fig. S5).

Many resistance factors appear regularly in the accessory genome of *E. faecium* (present in >15% of isolates) while rarely being detected in *E. lactis* (<15% of isolates). These include the vancomycin resistance conferring *vanA* and *vanB* operons, tetracycline resistance genes *tet(L*) and *tet(M)*, as well other detected genes and point mutations conferring resistance to streptomycin, trimethoprim, macrolides, quinolones, beta-lactams, lincosamides, streptogramin and aminoglycosides (Table S1). In addition to these resistance genes, eight virulence factors were commonly detected in *E. faecium* and not *E. lactis* (Table S2). These flagged virulence factors include the multiple adhesion and biofilm formation-associated genes *capD*, *ecbA*, *esp*, *fms15* and *prpA*. Additionally, two TIR domain containing genes that have been shown to promote enhanced proliferation and survival in blood [52] as well as *ptsD* which is a marker for a phosphotransferase system important for carbohydrate transport and intestinal colonization [53] appear in this group. Collectively, these acquired virulence factors in *E. faecium* can lead to increased host colonization and infection capabilities.

### Isolates with the highest levels of genomic identity between *E. faecium* and *E. lactis* do not show evidence of cross-species recombination

ANI values were used as a measure to select the isolates with the highest inter-species identities. Sequences with at least 20% of inter-species ANI above the 95% speciation threshold were identified. This group included 6 B1 *E. lactis*, 8 A1 *E. faecium* and 76 non-A1 *E. faecium* isolates. This selection method was chosen to include inter-species ANI values above the 95% threshold as well as many slightly below it.

As these samples were primarily non-A1 *E. faecium*, they were analysed with Gubbins alongside the *E. lactis* population to predict core genome recombination events. In this filtered population, no cross-species recombination events were detected.

### Some plasmids show evidence of mobilization across the species boundary

The effects of plasmid-associated content on the observed ANI values were also analysed. The whole-genome ANI values of the selected isolates were compared with those computed using only chromosome-associated contigs. Plasmid-associated contigs were identified for removal using the mlplasmids *E. faecium* model, as well as a newly trained *E. lactis* model (Section S3) [41]. The difference between whole-genome ANI and chromosome-associated ANI showed higher inter-species ANI when including plasmid-associated contigs (*P*=0.0001) with a mean difference of 0.00457% identity. This consistent difference in ANI suggests partially overlapping plasmidome content between *E. faecium* and *E. lactis* isolates with high identity. This contradicts slightly with observations from Arredondo-Alonso *et al*. who conclude that *E. faecium* plasmid content is largely source and clade specific [4]. The slight plasmid-associated ANI increase we observe may be driven by genes commonly found across multiple plasmids, such as those involved with replication and mobilization.

To study this further, the plasmid replicons in each assembly were screened with PlasmidFinder. The most common replicon type detected was *repUS15* (RepA_N) across both *E. faecium* and *E. lactis* as it was detected in at least 79% of the isolates of each clade (Figs S6 and S7). We detected this replicon type on plasmid-associated contigs often larger than 100 kb, meaning they represent a large amount of mobilizable genetic material across the species boundary. Most other detected plasmid replicons, with the exception of *rep1*, appear to correspond somewhat with phylogenetic clustering (Fig. S6).

## Discussion

As a comparatively more benign *E. lactis* is less studied and suffers from a lack of publicly available data. Using a novel dataset that includes large amounts of non-clinical *E. faecium* and *E. lactis*, we were able to study *E. lactis* genome content and population structure more thoroughly than previous studies [8,10,13, 14, 18]. Notably, genomes from the Norwegian marine collection and the Tromsø7 study account for 371 of the 454 *E. lactis* isolates in our study population. The inclusion of these environmental and human commensal enterococcal genomes is vital in the analysis done in this study.

With the large numbers of non-clinical *E. lactis* genomes, we were able to identify a unique clade within *E. lactis*, which we named B1. Additionally, we were able to confirm with a higher confidence that *E. lactis* meets ANI-based criteria for being distinguished as a species distinct from *E. faecium*. The reclassification of clade B to *E. lactis* is not yet a fully accepted paradigm in the literature [54,55]. Schwartzman *et al*. studied a variety of enterococcal species and used previously sequenced isolates as reference for phylogenetic and diversity locus analyses. Their selected *E. faecium* type strain for diversity locus analysis and *E. lactis* representative in phylogenetic analysis, E1007, was also included in our collection under the alias LE0048 and belongs to clade B1 *E. lactis*. For the purposes of identifying novel enterococcal species, grouping *E. lactis* and *E. faecium* is unlikely to obscure important findings. However, in this study, we show that separating these species allows for a more comprehensive investigation of the less clinically relevant *E. lactis*.

Higher allelic diversity in *E. lactis* core genes indicates it as a more genomically diverse species than *E. faecium*. This observation is statistically significant despite the fact that 70% of the *E. lactis* isolates in this study were isolated from a representative adult population in Tromsø municipality of Norway in 2015. The low geographic and temporal diversity of the *E. lactis* sample population likely biases the allelic diversity values computed in this study, particularly when comparing to a more globally dispersed *E. faecium* population. A globally distributed *E. lactis* population would thus be expected to show higher core genome diversity.

The observed differences in core genome diversity must be driven by mechanisms other than recombination events. Without elevated levels of HGT in core genes, the observed diversity in *E. lactis* is either driven by external diversifying pressures or temporally dependent mutation. External diversifying pressures from variance in lifestyle of the human hosts such as diet and exercise may contribute to the allelic diversity observed in non-hospitalized adult gut flora samples [56,60]. Isolates found in hospitalized human hosts would face a different set of selection pressures, such as the presence of antimicrobials. These differences in environmental pressures can be partially accounted for by comparing the DI values of *E. lactis* to non-A1 *E. faecium*. Both of these groups contain comparable fractions sourced from hospitalized humans, but non-A1 *E. faecium* has a much more diverse array of host species, which would theoretically apply diversifying pressures on the population. Consequently, the most plausible explanation for the higher core genome allelic diversity in *E. lactis* represents a population that has occupied its niche and host population for a longer period of time than *E. faecium* has. Extending this comparison to the two major *E. faecium* subpopulations describes non-A1 *E. faecium* as significantly older than clade A1, which is corroborated by other studies that used molecular clock methods [8,9].

Time-calibrated phylogenetic analyses of the within- and between-species variation were challenging due to both *E. lactis* and *E. faecium* exhibiting high rates of homologous recombination. The fraction of the genome unaffected by detected recombination events proved too small to allow for robust dating of either the clades within species or the speciation event (Section S4). Additionally, the majority of *E. lactis* samples in this data collection come from the Tromsø7 study conducted over 2015 and 2016. The lack of temporal variation in the *E. lactis* population thus leaves little to no signal for calibrating molecular clock models.

The inferred temporal relationship between the two species mirrors that of A1 and the paraphyletic non-A1 group of *E. faecium*. With this as context, the increased host diversity in non-A1 *E. faecium* suggests this evolutionary divergence may have been driven by a need to colonize a wider variety of hosts.

A recent study including 2,714 *E. faecium* and 111 *E. lactis* genomes outlined similarities and differences in metabolic pathways between these species and observed that both species have a high abundance of carbohydrate metabolism and transport genes [14]. The sets of species-specific core genes found in our study include several carbohydrate metabolism factors – *xly* in *E. lactis* and *chbG*, *glxK*, *malH* and *nagE* included in *E. faecium*. The core *E. lactis* gene *xly* encodes xanthan lyase, an enzyme involved in the degradation of xanthan, a complex polysaccharide commonly found in food, cosmetics and pharmaceuticals [61,63]. Xanthan is also naturally produced by several species of plant-associated *Xanthomonas* [64]. These xanthan-producing bacteria are known to infect agricultural crops [65] and thus may have been present in human diets for a long time. The relative abundance of these genes in the species-unique core and soft core genes suggests the potential importance of carbohydrate supply and metabolism in the divergence of *E. lactis* and *E. faecium*. This aligns with previous research concluding that enterococcal speciation is partially driven by energy source availability [49].

Modelling of accessory genome content shows HGT trends that do not align with those seen in core genes. *E. faecium*, clade A1 in particular, displayed higher rates of accessory-associated HGT despite exhibiting lower rates of homologous recombination in the core genome. This contrast highlights the reliance A1 *E. faecium* has on MGEs for facilitating HGT. This result is congruent with previous research showing the importance of plasmids in the emergence and spread of resistance in *E. faecium* [4,8, 10, 14, 18].

The most common plasmid observed in each clade of the study population was a highly prolific megaplasmid *repUS15* (repA_N). Interestingly, megaplasmids of this plasmid type carrying multiple resistance genes and/or virulence factors are often found in clade A1 *E. faecium* and are shown to be conjugative [66,67]. Rare occurrences of such plasmids encoding vancomycin resistance in *E. lactis* [68] or a potent pore-forming toxin in *E. faecium* isolated from animals [69] have also been described. Therefore, the genes carried by such megaplasmids may be partially species specific. Despite this, it has been shown that this plasmid is capable of transferring chromosomal DNA between *E. faecium* and *E. lactis* [70]. Further study and analyses would be required to better understand the genomic content being transferred by these plasmids across species boundaries in order to reconcile our results with those from Arredondo-Alonso *et al*. [4].

The differences we observed in the resistomes and virulomes of *E. lactis* and *E. faecium* have already been studied and documented earlier [10,14, 18, 71]. The lower prevalence of AMR and virulence factors, especially acquired AMR and virulence factors, indicate that *E. lactis* strains could represent safer probiotic options than known *E. faecium* strains [53,72]. Recognizing the distinction between these species would help guide the design of future probiotics.

*E. lactis*, non-A1 *E. faecium* and A1 *E. faecium* display a trend of increasing rates of HGT corresponding to changes in host adaptation. Previous studies estimate that clade A1 diverged from non-A1 *E. faecium* ~500 years ago, while *E. faecium* and *E. lactis* split roughly 3,000 years ago [8,9]. A1 *E. faecium* displayed much higher rates of HGT in the accessory genome than non-A1 *E. faecium*. A similar relationship is seen comparing non-A1 *E. faecium* to *E. lactis*. This illustrates a trend in *E. faecium* diverging from *E. lactis* as a group with a much more mobile accessory genome, possibly due to environmental pressures. Non-A1 *E. faecium* is the most prevalent subpopulation in animal hosts and has the highest host diversity, which may be a driving factor for a more mobile and adaptable accessory genome when compared with *E. lactis*. A1 *E. faecium* has been shown to contain specific restriction modification system variants and a lack of CRISPR-Cas that can explain the increased acceptance of plasmids and other MGEs [4,8, 73].

In summary, core gene diversity and CIs indicate that *E. lactis* represents an older population than *E. faecium*. Isolation source diversity of individual subclades suggests that the original evolutionary split may have been driven by the transfer of a common ancestor from human to multiple human-associated animal hosts, i.e. pets and domesticated animals, each of which typically have host-specific diets that can drive adaptation of gut bacteria through selective pressures on metabolic traits. Our study also highlights the importance of sampling less clinically relevant and pathogenic bacteria to improve understanding of the evolution of these bacterial populations and their interplay with pathogenic species.

## Supplementary material

10.1099/mgen.0.001420 Uncited Table S1.

10.1099/mgen.0.001420 Uncited Table S2.

10.1099/mgen.0.001420 Uncited Supplementary Material 1.

## Abbreviations

AMR: antimicrobial resistance
ANI: average nt identity
AST: antimicrobial susceptibility test
CI: consistency index
COG: clusters of orthologous genes
DI: diversity index
DoF: degrees of freedom
HGT: horizontal gene transfer
MGE: mobile genetic element
NCBI: National Center for Biotechnology Information
RF: random forest
rMLST: ribosomal multilocus sequence typing
SVM: support vector machine
