# Supplementary material for: Enterococcus lactis is ecologically and genetically distinct from the major opportunistic pathogen Enterococcus faecium
Source: Microb Genom. 2025 Jun 6;11(6):001420. doi: 10.1099/mgen.0.001420 (PMC12144320; doi:10.1099/mgen.0.001420)
Supplement: Uncited Supplementary Material 1. [file mgen-11-01420-s003.pdf]

Enterococcus lactis is the likely progenitor of the major opportunistic pathogen *Enterococcus faecium*

Theodor A. Ross, Jessin Janice, Sergio Arredondo-Alonso, Iren H. Löhr, Einar Holsbø, Jukka Corander, Anna K. Pöntinen, Michael Kampffmeyer, Kristin Hegstad

Supplementary:

1. Supplementary Figures
2. Species-specific core COGs
3. Novel *E. lactis* mlplasmids model successfully predicts plasmid contigs
4. Failure of calibrated temporal evolution analysis
5. Supplementary Methods

1. Supplementary Figures

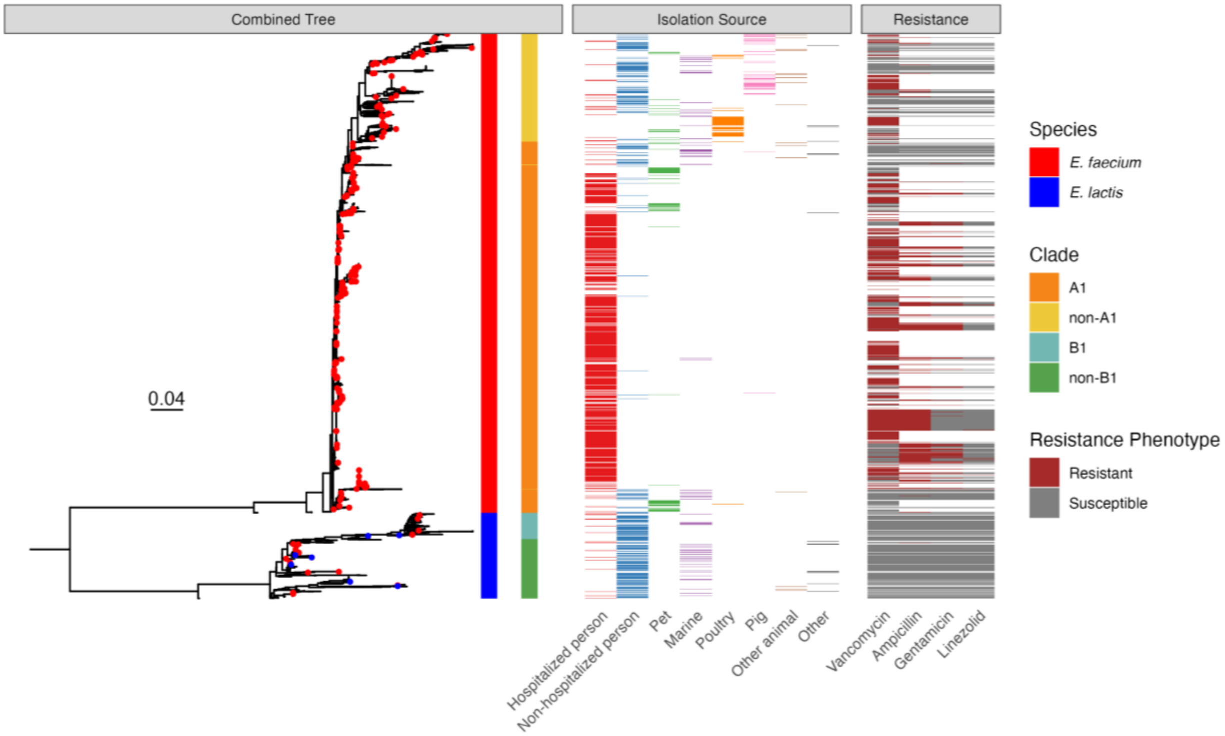

Supplementary Figure S1. Approximate maximum likelihood phylogeny computed with FastTree. The colorbars in the Combined Tree panel display the species and clade of the corresponding isolates. The colored leaf tips correspond to circularized assemblies collected from NCBI. The isolation source and phenotypic resistances are displayed for all isolates that had such data available. Elac=*E. lactis*, Efm=*E. Faecium*.

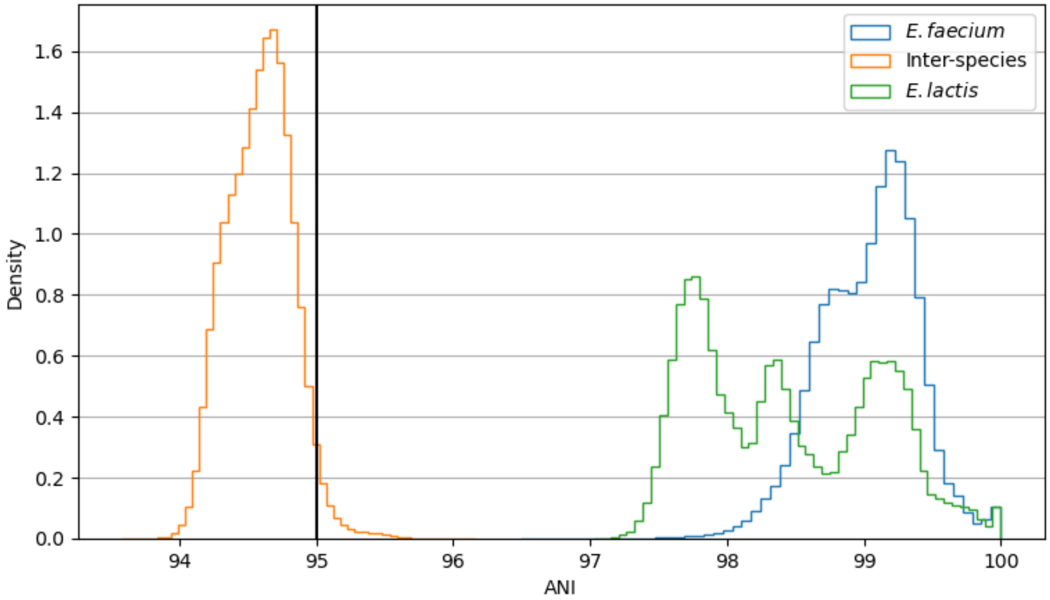

Supplementary Figure S2. Histogram of ANI values between all isolate pairs in the study population. All three distributions (Inter-species, *E. faecium* intra-species, and *E. lactis* intra-species) are compared to the 95 percent speciation threshold, marked in black.

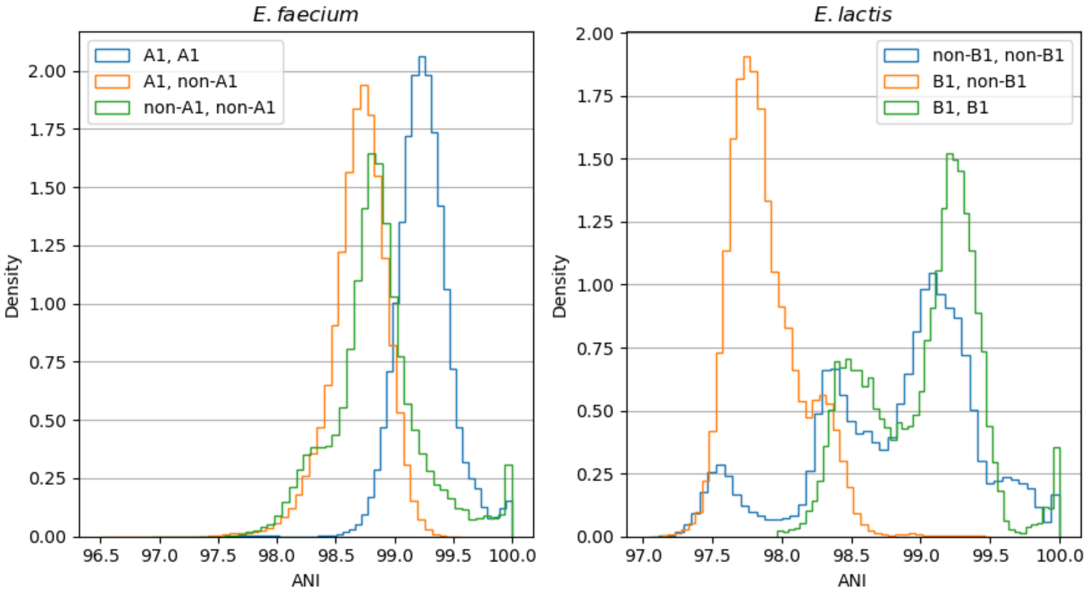

Supplementary Figure S3. Histograms of intra-species ANI values separated by clade relations within each species.

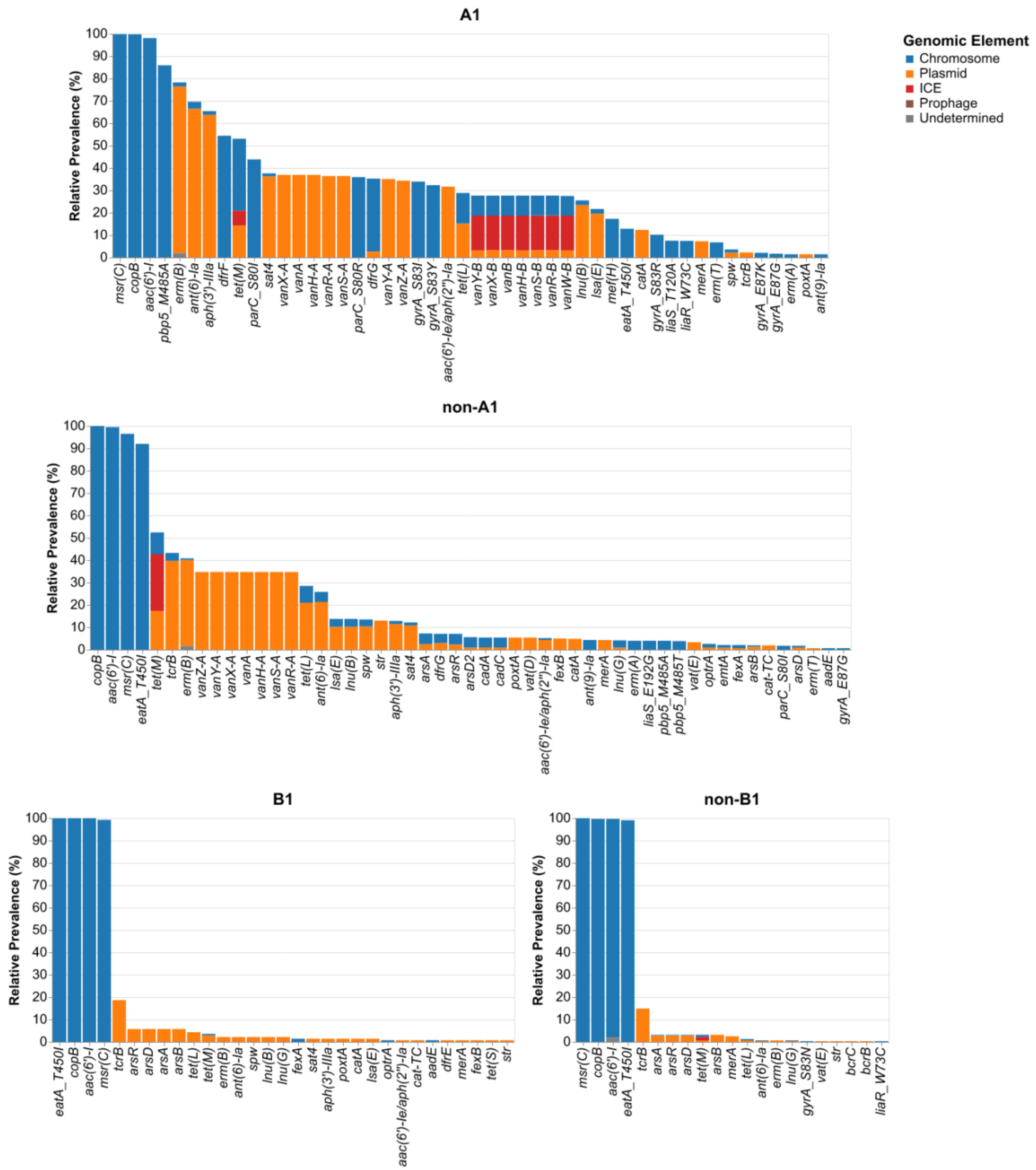

Supplementary Figure S4. Relative prevalence of detected resistance factors in each subpopulation described in this study. Only the 50 most commonly detected AMR factors were included in the *E. faecium* figure; all AMR factors detected in *E. lactis* are shown here. Colors describe the genomic elements the factors were detected on; factors detected on contigs shorter than 1000 bp are listed as "Undetermined".

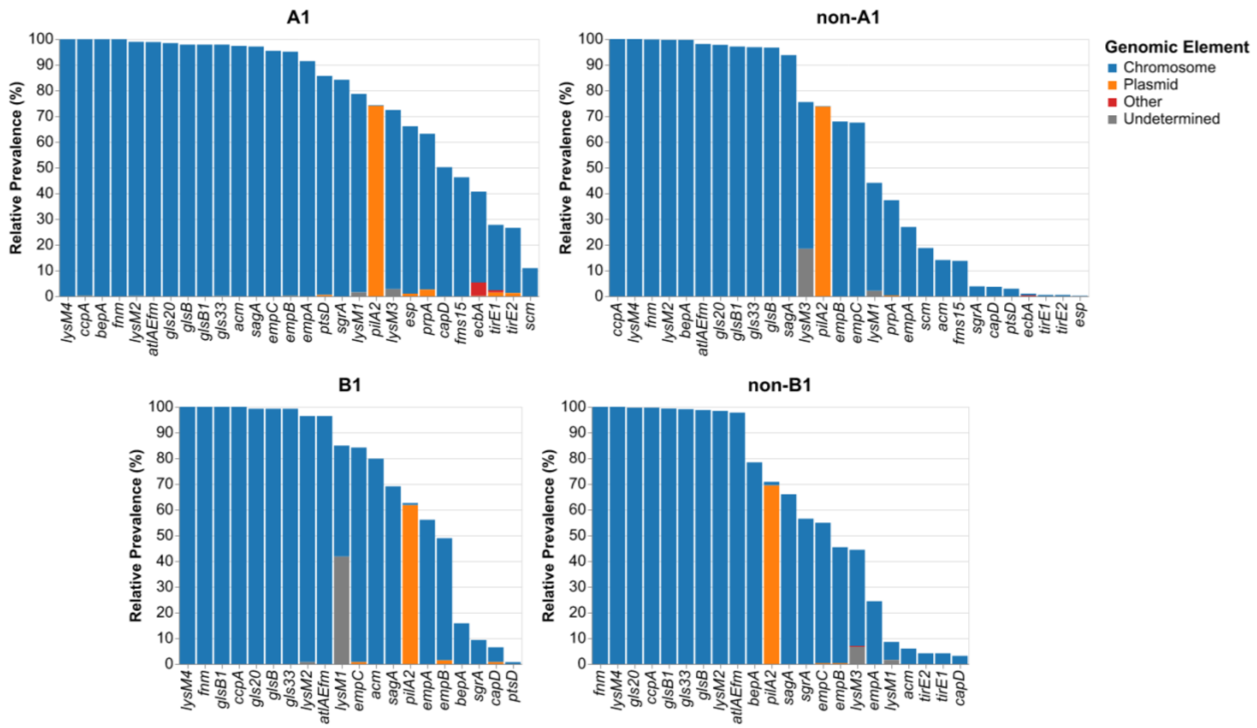

Supplementary Figure S5. Histograms describing the relative prevalence of known enterococcal virulence factors detected in each subpopulation. Colors correspond to the relative fractions of the category of genomic element each factor was detected on. Factors detected on phages and ICEs were grouped together as “Other” for visual clarity. Factors found on contigs under 1000 bp were reported as “Undetermined”.

2. Species-specific core COGs

Below are the annotated unique core COGs detected in the *E. faecium* population and their putative functions. Sequence information for these and the unique core hypothetical COGs are listed in supplementary file Efm_Elac_unique_core_COGS.xslx where the most common allele for each COG is reported.

- *ybiT_2; expZ; ybiT_3; ybiT_1*
  - Putative ABC transporter ATP-binding protein YbiT
  - **(expZ) Nucleotide binding protein**
- *chbG; chbG_1; chbG_2*
  - Chitin disaccharide deacetylase
- *lnrK*
  - Transcriptional regulatory protein LnrK
- *glxK_1; glxK_2; glxK*
  - Glycerate kinase
- *simA; malH; malH_1; aglB; malH_2*
  - Maltose-6'-phosphate glucosidase MalH
- *desK*
  - Two-component sensor histidine kinase DesK
- *nagE; nagE2; gamP_2; gamP; nagE_2; nagF; malP_3; gamP_1; ptsG_2; malP_1; ptsG_4; nagE_1*
  - N-acetylglucosamine-specific PTS enzyme
  - Putative PTS system glucosamine-specific EIICBA component
  - Maltose phosphorylase
  - PTS system glucose-specific EIICBA component
- *yhaX; ycsE*
  - Stress response protein
- *ybhF; lnrL; cysA; cysA_2*
  - Putative multidrug ABC transporter ATP-binding protein YbhF
  - Linearmycin resistance ATP-binding protein LnrL
  - Sulfate/thiosulfate ABC transporter ATP binding subunit

Below are the annotated unique core COGs detected in the *E. lactis* population and their putative functions. Sequence information for these and the unique core hypothetical COGs are listed in supplementary file Table 2 where the most common allele for each COG is reported.

- *coiA*
  - Competence protein
- *xly*; *xly_1*; *xly_2*
  - Xanthan lyase

Supplementary Table S1. AMRFinderPlus results detected as shell genes in *E. faecium* (present in >15% of isolates) and as cloud genes in *E. lactis* (present in <15% of isolates).

**Gene**	**Class**	**Protein involved**
vanA gene cluster	GLYCOPEPTIDE	Vancomycin resistance proteins VanA, VanH-A, VanR-A, VanS-A, VanX-A, VanY-A, and VanZ-A
vanB gene cluster	GLYCOPEPTIDE	Vancomycin resistance proteins VanB, VanH-B, VanR-B, VanS-B, VanW-B, VanX-B, VanY-B
aac(6')-Ie/aph(2")-Ia	AMINOGLYCOSIDE	bifunctional aminoglycoside N-acetyltransferase AAC(6')-Ie/aminoglycoside O-phosphotransferase APH(2")-Ia
ant(6)-Ia	AMINOGLYCOSIDE	aminoglycoside nucleotidyltransferase ANT(6)-Ia
aph(3')-IIIa	AMINOGLYCOSIDE	aminoglycoside O-phosphotransferase APH(3')-IIIa
dfrF	TRIMETHOPRIM	trimethoprim-resistant dihydrofolate reductase DfrF
dfrG	TRIMETHOPRIM	trimethoprim-resistant dihydrofolate reductase DfrG
erm(B)	MACROLIDE	23S rRNA (adenine(2058)-N(6))-methyltransferase Erm(B)
gyrA_S83I / *gyrA_S83Y*	QUINOLONE	DNA gyrase subunit A GyrA with S83I / S83Y mutation
lnu(B)	LINCOSAMIDE	lincosamide nucleotidyltransferase Lnu(B)
lsa(E)	LINCOSAMIDE / STREPTOGRAMIN	ABC-F type ribosomal protection protein Lsa(E)
parC_S80I / *parC_S80R*	QUINOLONE	DNA topoisomerase IV subunit A ParC with S80I / S80R mutation
pbp5_M485A	BETA-LACTAM	penicillin-binding protein 5 with M485A mutation
sat4	STREPTOTHRICIN	streptothricin N-acetyltransferase Sat4
tet(L)	TETRACYCLINE	tetracycline efflux MFS transporter Tet(L)
tet(M)	TETRACYCLINE	tetracycline resistance ribosomal protection protein Tet(M)

Supplementary Table S2. Virulence factors detected with ABRicate that are found as shell genes in *E. faecium* (present in >15% of isolates) and cloud genes in *E. lactis* (present in <15% of isolates).

**Gene**	**Product - function**
capD	Capsular polysaccharide biosynthesis protein adhesion - avoid opsonic killing
ecbA	Collagen binding MSCRAMMM - adhesion
esp	Enterococcal surface protein - biofilm formation (conserved sequence)
fms15	*E. faecium* surface protein of the MSCRAMM family - adhesion
prpA	Prolin rich protein A - bind to the extracellular matrix proteins fibrinogen and fibronectin
ptsD	Phosphotransferase system subunit IID - intestinal colonization determinant during antibiotic treatment
tirE1	TIR-domain containing protein - promotes survival in blood
tirE2	TIR-domain containing protein - promotes survival in blood

3. Novel *E. lactis* mlplasmids model successfully predicts plasmid contigs

The mlplasmids toolkit includes species-specific machine learning based models for prediction of plasmid or chromosome association of contigs. At the time of development, the publicly available package included a model for *E. faecium*, but not for *E. lactis*. Therefore we trained an mlplasmids model using the circularized *E. lactis* PacBio long-read sequences (n=67) in our study population.

The novel *E. lactis* mlplasmids model achieved comparable performance to the existing mlplasmids models. The support-vector machine (SVM) model displayed the best performance with the withheld set of validation contigs (accuracy=0.90, F1-score=0.77). Performance improved further after removing contigs shorter than 1000bp (accuracy=0.94, F1-score=0.86). Similar to the existing mlplasmids models, we noted higher performance metrics when using nucleotides as the unit of measurement. This is likely driven by higher error rates for shorter contigs. Performance summaries with respect to the 1463 test contigs for each of the five models trained are shown in Supplementary Figure S4. The *E. lactis* trained SVM outperformed the *E. faecium* trained mlplasmids model when used in predicting *E. lactis* contig association.

The newly trained *E. lactis* model has been integrated into the publicly available mlplasmids package https://gitlab.com/sirarredondo/mlplasmids.

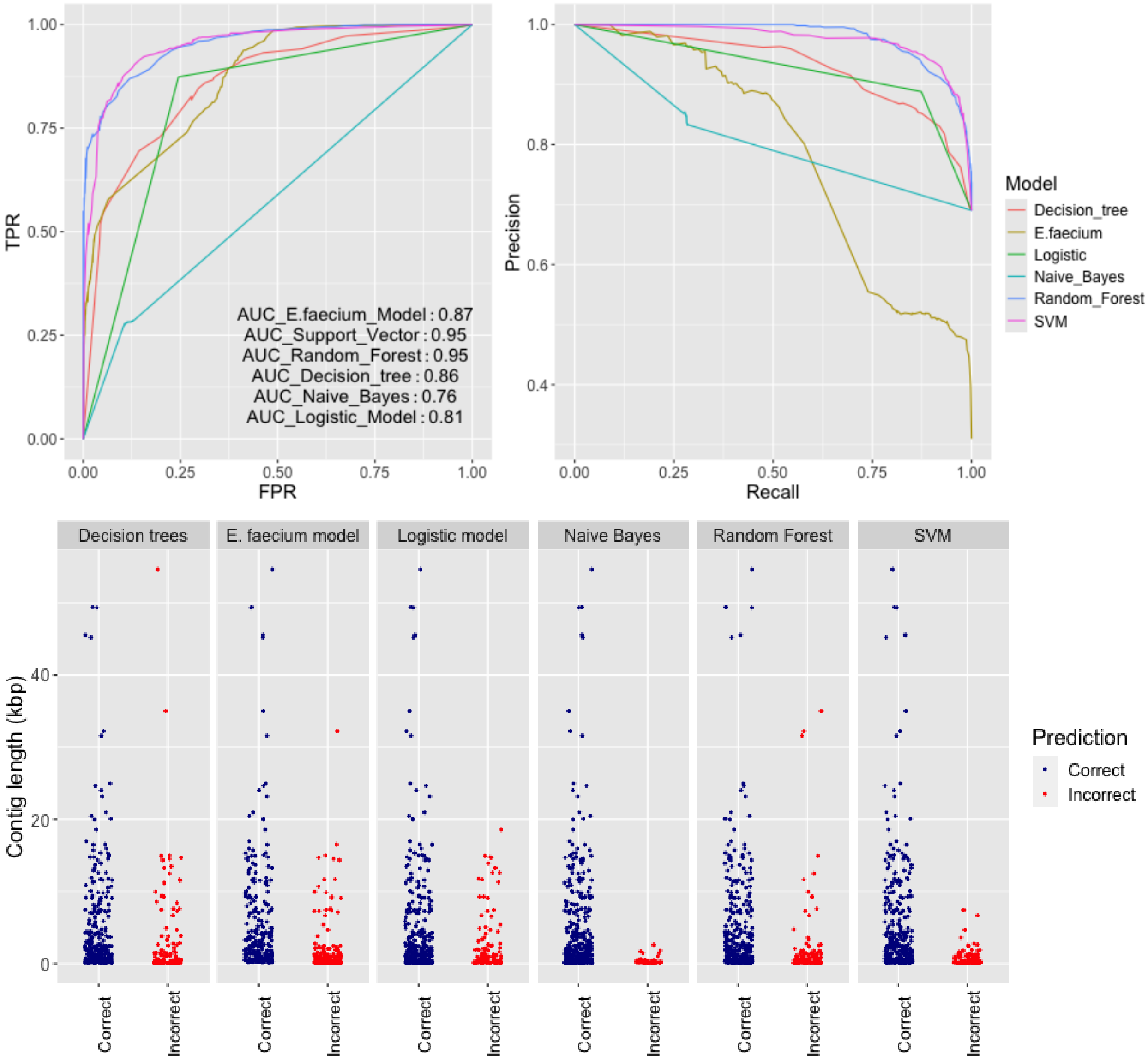

Supplementary Figure S6. (Top-left) Receiver operating characteristic (ROC) curves for the trained models for predicting replicon association of *E. lactis* contigs. (Top-right) Precision-recall curves for trained models predicting replicon association of *E. lactis* contigs. (Bottom) Visualization of the effect of contig length on classification accuracy of the trained models.

4. Failure of calibrated temporal evolution analysis

In an attempt to estimate the time of divergence between clades and species, a temporal analysis was attempted using recombination masked genomes. A subset of *E. faecium* isolates were selected alongside all of the available *E. lactis* genomes for recombination detection using Gubbins (v3.3.5). A temporal model of the species evolution and mutation rates were then

estimated using TempEst (v1.5.3). The best model achievable reached an r-squared value of 3.57×10^{-2} and a correlation coefficient of 0.189. Consequently, the resulting model was not used for further analysis or inference.

5. Supplementary Methods

Gene selection analysis

Within each population, the top and bottom 2.5% of the DI values were isolated to mark the genes under diversifying and purifying selection, respectively. To accomplish this, a beta distribution was fit to the distribution of DI values for each population using maximum likelihood estimation (MLE). Because the DI values are necessarily bound between 0 and 1, the location and scale parameters were held to 0 and 1, leaving only the shape parameters to be optimized. After fitting the beta distribution, the genes belonging to the top and bottom 2.5 percentiles were isolated.

ICE detection

We screened the genome assemblies for integrative conjugative elements (ICEs) by BLAST against the ICEberg 2.0 database [1] (downloaded on October 2nd, 2023), using ABRicate (v1.0.1) with minimum coverage and identity thresholds both set to 80 percent [2. This threshold was chosen to allow for a higher likelihood of detecting ICEs that may be missing a gene when compared to a sample in the ICEberg database.

Phage detection

Phage elements in each genome were screened for using geNomad (v1.7.1) [3], with score calibration enabled and otherwise default parameters. Detected phage elements were then filtered to keep only those with a phage prediction score over 0.9, greater than 2 genes within the predicted phage element, and at least one hallmark virus sequence.

Works Cited

[1] M. Liu, X. Li, Y. Xie, D. Bi, J. Sun, J. Li, C. Tai, Z. Deng and H. Y. Ou, "ICEberg 2.0: an updated database of bacterial integrative and conjugative elements," *Nucleic Acids Research,* vol. 47, no. D1, pp. D660-D665, 8 1 2019.

[2] T. Seemann, "Abricate," [Online]. Available: https://github.com/tseemann/abricate.

[3] A. P. Camargo, S. Roux, F. Schulz, M. Babinski, Y. Xu, B. Hu, P. S. G. Chain, S. Nayfach and N. C. Kyrpides, "Identification of mobile genetic elements with geNomad," *Nature Biotechnology,* vol. 42, no. 8, pp. 1303-1312, 2023.
